# The prevalence of seat belt use among drivers and passengers: a systematic review and meta-analysis

**DOI:** 10.1186/s42506-023-00139-3

**Published:** 2023-08-02

**Authors:** Shiva Kargar, Alireza Ansari-Moghaddam, Hossein Ansari

**Affiliations:** Health Promotion Research Center, Zahedan University of Medical Sciences, Zahedan, Iran

**Keywords:** Prevalence, Seat belt usage, Drivers, Passengers, Meta-analysis

## Abstract

**Background:**

Seat belts might save people’s lives in car accidents by preventing severe collision damage and keeping passengers safe from critical injuries. This meta-analysis was performed to assess the prevalence of seat belt use among drivers and passengers.

**Methods:**

The databases of PubMed, Web of Science (WOS), and Google Scholar were searched from the beginning of 2000 to late December 2020 to identify studies that investigated the prevalence of seat belt use among drivers and passengers. The pooled prevalence was calculated using a random-effects model. The STATA-v14 software was used to perform data analysis.

**Results:**

Sixty-eight studies that met the inclusion criteria and were suitable for this meta-analysis were identified. The pooled prevalence of seat belt use was 43.94% (95% *CI*: 42.23–45.73) among drivers, 38.47% (95% *CI*: 34.89–42.42) among front-seat passengers, and 15.32% (95% *CI*: 12.33–19.03) among rear-seat passengers. The lowest seat belt use among drivers and passengers was observed in Asia, the Middle East, and Africa, while the highest use was reported in Europe and America. Moreover, the prevalence of seat belt use was higher among women drivers [51.47% (95% *CI*: 48.62–54.48)] than men drivers [38.27% (95% *CI*: 34.98–41.87)] (*P* < 0.001). Furthermore, the highest prevalence of seat belt use was seen among drivers (68.9%) and front-seat passengers (50.5%) of sports utility vehicles (SUVs); in contrast, the lowest prevalence was observed among drivers and passengers of public vehicles such as buses, minibuses, and taxis.

**Conclusions:**

In general, the prevalence of seat belt use was not high among drivers and was even lower among passengers. Moreover, drivers and passengers in Asia, the Middle East, and Africa had the lowest prevalence of seat belt usage. Additionally, drivers and passengers of public transportation (buses, minibuses, and taxis) had a lower rate of seat belt use, especially among men. Therefore, effective interventional programs to improve seat belt use should be designed and implemented, particularly among these at-risk populations in Asia, the Middle East, and Africa.

**Supplementary Information:**

The online version contains supplementary material available at 10.1186/s42506-023-00139-3.

## Introduction

Motor vehicle crashes are one of the significant causes of morbidity and mortality worldwide [1]. According to the World Health Organization, around 1.3 million people lose their lives every year, and between 20 and 50 million are injured as a result of road traffic accidents [2].

Road injuries create an enormous economic burden for countries. The global economy is estimated to incur a cost of US $1.8 trillion (constant 2010 US dollars) due to road injuries in 2015–2030, equivalent to 0.12% of the global gross domestic product (GDP) annually [3]. As a result, it is crucial to establish motor vehicle crashes prevention programs worldwide.

Most deaths from motor vehicle crashes occur among the car’s occupants [4]. Therefore, seat belts are a cost-effective preventive measure for reducing the severity of injury, disability, and death caused by road accidents [5]. By wearing a seat belt, the risk of death among drivers and passengers in the front seat decreases by 45–50%, and the risk of death and serious injury among passengers in the rear seat decreases by about 25% [2].

The prevalence of seat belt use varies across different countries of the world and depends to some extent on the regulations in place. Seat belt use in low- and middle-income countries is not high, despite strict driving laws and fines for those who do not use seat belts [6, 7]. Studies have shown that men, young people (18–34 years old), obese individuals, rear seat occupants, and villagers have lower rates of seat belt use [8–10].

Accordingly, estimating the prevalence of seat belt use is very important for public health policymakers to implement programs aiming at reducing mortality and morbidity from motor vehicle crashes. Moreover, there were no recent systematic reviews on seat belt use in the previous 5 years. Therefore, this study was conducted to determine the prevalence of seat belt use among drivers, front-seat passengers, and rear-seat passengers from the beginning of 2000 to late December 2020.

## Methods

### Search strategy and study selection

1. This meta-analysis was performed according to the Preferred Reporting Items for Systematic Reviews and Meta-Analyses (PRISMA) guidelines [11]. Relevant articles were identified through the databases of PubMed, Web of Science (WOS), and Google Scholar from the beginning of 2000 to late December 2020 using combined keywords and Medical Subject Headings (MESH) heading strategies such as "Seatbelt, "Safety belt, "Seat Belt Usage, "Prevalence," "Frequency, "Driver," "Vehicle," and "Passenger." Additionally, references from previous reviews were scanned to identify other relevant articles.

Studies that met the following criteria were selected for the current meta-analysis:

1. Cross-sectional studies that reported data on seat belt use in commercial or personal vehicles for both drivers and front or rear seat passengers separately.
2. Studies performed on passengers over the age of 12, as the seat belt use is suggested for those aged 12 and above, and passengers under the age of 12 should use child restraints [12].
3. The target population was either the general population or a specific population, such as high school and university students, drivers, or passengers involved in motor vehicle crashes.

The exclusion criteria were as follows:

1. Studies that were not written in English
2. Studies conducted on specific populations, such as pregnant women and people with physical limitations on seat belt use and those who had undergone abdominal surgery
3. Studies that lacked data for prevalence calculation with a 95% confidence interval
4. Studies that reported mixed reporting of seat belt use among drivers and passengers

We also excluded studies for which we were unable to obtain a full text after contacting the corresponding author.

### Quality assessment

An assessment of the quality of the included studies was performed using a quality checklist adapted from Loney et al. [13]. The checklist assesses the different aspects of methodology (study design and sampling method, sampling frame, sample size, appropriate measurement, unbiased measurement, and response rate) as well as the interpretation of results and application of findings. The study received 1 point for each criterion that was met. Scores for high-quality studies range from 7 to 8 points, for moderate-quality studies from 4 to 6 points, and for low-quality studies from 0 to 3.

### Data extraction

Two researchers independently screened the identified articles from the databases based on the title and abstract. Then, the full text and abstract of the related articles were reviewed. Data were extracted from the eligible articles and recorded in an Excel checklist containing the names of authors, year of publication, study setting (country), age, gender, the number of participants, method, the prevalence of wearing a seat belt, and its 95% confidence interval. In this study, individuals who reported that they always, sometimes, often, full time, most of the time, or regularly wear seat belts were considered seat belt users. Table 1 presents a summary of the included studies in the meta-analysis.

**Table 1:** Summary of the studies included in the meta-analysis

**Authors**	**Year of publication**	**Country**	**Setting**	**Sampling**				Driver		**Front seat passengers**	**Rear seat passengers**	**How often**
				**Age**	**Method**	**Sex**	***N***		**Prevalence (95% *CI*)**	**Prevalence (95% *CI*)**	**Prevalence (95% *CI*)**	
Kim [14]	2003	Hawaii		≥ 18	_	FM	3457	FM	77.5 (76.1–78.9)	78.2 (75.5–80.9)	_	_
La [15]	2013	Vietnam	Hanoi	31.9 (6.8)	_	FM	1214	FM	50.7 (47.8–53.5)	_	_	Always
								FM	11.7 (10.0–13.7)	_	_	Usually
								FM	10.7 (9.10–12.6)	_	_	Sometimes
Routley [16]	2009	China	Nanjing	_	Interview survey	FM	234	FM	56.4 (49.8–62.9)	_	_	Always/mostly
								FM	20.5 (15.5–26.3)	_	_	Sometimes
					Roadside observation	FM	9294	FM	31.7 (30.7–32.6)	_	_	_
					In-taxi observation	FM	285	FM	20.4 (15.8–25.5)	_	_	_
Mohammadi [17]	2011	Iran	Kerman	18–24	_	FM	250	FM	18.0 (13.4–23.3)	_	_	Always
								FM	1.06 (11.6–21.1)	_	_	Most of the time
								FM	20.0 (15.2–25.50)	_	_	Sometimes
						M	179	M	10.6 (6.05–16.0)	_	_	Always
								M	15.6 (10.6–21.8)	_	_	Most of the time
								M	19.9 (14.0–26.1)	_	_	Sometimes
						F	71	F	36.6 (25.5–48.9)	_	_	Always
								F	16.90 (9.0–27.6)	_	_	Most of the time
								F	21.1 (12.3–32.4)	_	_	Sometimes
Allena [18]	2019	Virginia		≥ 65	Stratified sampling	FM	751	FM	96.4 (94.8–97.6)	_	_	Always
						M	392	FM	2.10 (1.20–3.40)	_	_	Nearly always
						F	350	FM	0.50 (0.10–1.30)	_	_	Sometimes
Pérez-Núñez [19]	2013	Mexico		≥ 10	Randomly, observational	FM	12,064	FM	45.0 (44.3–45.7)	_	_	_
García-España [20]	2012	USA		_	Randomly selected	FM	3126	FM	81.5 (77.0–85.2)	68.9 (64.5–72.9)	_	Often/always
Chen [21]	2015	USA		47.8		FM	1265	FM	86.1 (81.6–90.7)	_	_	Often
						M	1183	FM	7.80 (6.50–9.10)	_	_	Sometimes
						F	86			_	_	-
Bener [22]	2013	Qatar		≥ 20	Multistage stratified cluster	FM	1824	FM	17.6 (15.9–19.4)	_	_	Always
								FM	29.6 (27.5–31.8)	_	_ _ _	More than half of the trip
						M	1362	M	17.3 (15.3–19.4)	_	_	Always
								M	28.7 (26.3–31.2)	_	_	More than half of the trip
						F	462	F	18.6 (15.1–22.4)	_	_	Always
								F	32.2 (28.0–36.7)	_	_	More than half of the trip
Kritsotakis [23]	2019	Greece		18–20	Random sample	FM	536	FM	72.1 (67.9–76.1)	72.1 (68.1–75.9)	19.7 (16.4–23.3)	Very often, regularly
								FM	15.4 (12.3–18.9)	20.6 (17.3–24.3)	23.3 (19.7–27.1)	Occasionally
						M	375	M	71.4 (66.4–76.1)	_	_	Very often, regularly
								M	17.8 (13.9–22.3)	_	_	Occasionally
						F	161	F	73.9 (65.7–81.0)	_	_	Very often, regularly
								F	9.40 (5.10–15.5)	_	_	Occasionally
Mohammadi [24]	2015	Iran	Sistan and Baluchistan	_	_	FM	1427	FM	58.2 (55.7–61.0)	73.3 (60.3–83.9)	_	_
						M	1393	M	58.3 (55.6–60.9)	32.3 (26.9–38.2)	_	_
						F	11	F	72.7 (39.0–93.9)	62.3 (51.1–72.6)	_	_
Popoola [25]	2013	Nigeria	Makurdi	_	Observational	FM	500	FM	57.0 (52.4–61.4)	40.3 (37.4–43.2)	3.00 (1.90–4.50)	_
						M	1137	M	31.2 (28.5–34.0)	_	_	_
						F	637	F	22.6 (19.4–26.0)	_	_	_
Sangowawa [26]	2010	Nigeria	Ibadan	_	Cluster sampling technique, observational	FM	5757	FM	31.7 (30.0–33.4)	10.3 (8.60–11.6)	0.40 (0.10–0.90)	_
						M	2627	M	30.2 (28.5–32.0)	_	_	_
						F	3130	F	47.3 (40.9–53.8)	_	_	_
Mohammadzadeh [27]	2015	Iran	Kashan		_	FM	822	FM	68.1 (64.8–71.3)	65.5 (58.2–72.3)	30.2 (20.2–41.8)	_
Praveen [28]	2020	India			Observational	FM	3345	FM	51.3 (49.6–53.1)	_	5.90 (5.00–6.80)	_
						M	3121	M	50.1 (48.4–51.9)	_	6.20 (5.00–7.50)	_
						F	224	F	63.8 (57.1–70.1)	_	5.60 (4.40–7.10)	_
Bener [29]	2008	Qatar		18–65	A multistage stratified cluster	FM	1110			_	_	
								FM	35.4 (32.5–38.3)	_	_	More than half of the trips
								FM	19.4 (17.1–21.9)	_	_	Always
						M	847	M	34.3 (31.1–37.6)	_	_	More than half of the trips
								M	18.3 (15.7–21.0)	_	_	Always
						F	263	F	38.7 (32.844.9)	_	_	More than half of the trips
								F	23.1 (18.2–28.7)	_	_	Always
Briggs [30]	2008	USA		≥ 16	Stratified three-stage cluster	FM	12,731	FM	59.0 (55.3–62.6)	_	_	Always
						M		M	52.1 (48.4–55.8)	_	_	Always
						F		F	66.7 (62.7–70.5)	_	_	Always
Fernandez [31]	2006	USA	Massachusetts	≥ 18	Systematic sampling	FM	381	FM	50.1 (45.0–55.2)	_	_	_
						M		M	42.0 (34.9–49.4)	_	_	_
						F		F	58.0 (51.3–65.8)	_	_	_
Alomari [32]	2020	Jordan	Amman, Irbid, Zarqa	≥ 18	Observational	FM	2098	FM	13.0 (11.3–13.7)	8.00 (6.00–9.20)	_	_
						M		M	9.90 (8.80–11.0)	6.80 (5.00–8.90)	_	_
						F		F	28.6 (24.5–33.1)	8.90 (6.20–12.2)	_	_
Gebresenbet [33]	2019	Ethiopia	Addis Ababa	May—55	Systematic sampling	FM	167	FM	59.2 (38.8–77.6)	_	_	_
						M	122		_	_	_	
						F	42		_	_	_	
Raman [34]	2014	Kuwait		≥ 18	_	FM	741	FM	41.5 (37.9–45.2)	30.4 (27.1–33.9)	_	Always
						M	415	FM	31.7 (28.3–35.2)	32.9 (29.5–36.4)	_	Mostly/sometimes
						F	325		_	_	_	_
Jermakian [35]	2018	USA		≥ 18	_	FM	1163	FM	_	_	72.1 (70.1–75.3)	Always
						M	_	M	_	_	67.7 (62.8–72.6)	
						F	_	F	_	_	75.4 (71.679.2)	
						FM	1163	FM	_	_	16.2 (14.1–18.5)	Part time
						M		M	_	_	17.4 (9.40–25.4)	
						F		F	_	_	15.3 (8.20–22.4)	
Koushki [36]	2006	Kuwait		≥ 18	Random sample	FM	1467	FM	36.6 (34.1–39.1)	_	_	Always
								FM	13.5 (11.8–15.3)	_	_	Often
						M	881	M	18.2 (15.7–20.9)	_	_	Always
								M	16.0 (13.6–18.6)	_	_	Often
						F	586	F	64.0 (60.1–68.0)	_	_	Always
								F	9.70 (7.40–12.4)	_	_	Often
Wong [37]	2016	Asia	Singapore, Malaysia, India, China	≥ 18	_	FM	4576	FM	91.4 (90.3–92.4)	87.4 (85.0–89.6)	44.7 (41.2–48.2)	_
Vaughn [38]	2012	USA	_	≥ 18	Multistage area probability	FM	75,782		_	_	_	
						M		M	97.2 (96.8–97.5)	96.85 (96.5–97.2)	_	_
						F		F	98.4 (98.1–98.8)	98.3 (98.0–98.6)	_	_
Taylor [39]	2019	USA	_	≥ 18	_	FM	5292	FM	_	68.0 (66.6–69.3)	63.0 (62.7–65.3)	Full time
						M	2465	M	_	_	64.0 (62.0–65.9)	Full time
						F	2796	F	_	_	62.9 (61.1–64.7)	Full time
Tavafian [40]	2011	Iran	Bandar Abbas	Mean 31.6 ± 8.7	Convenience	FM	251	FM	53.3 (47.0–59.6)	_	_	Often
								FM	32.6 (26.9–38.8)	_	_	Sometimes
Siviroj [41]	2012	Thailand	_	_	Quota sampling	FM	13,722	FM	71.7 (70.9–72.4)	_	_	_
						M	10,603	M	70.2 (69.3–71.0)	_	_	_
						F	3095	F	76.7 (75.2–78.2)	_	_	_
Densu [42]	2013	Ghana	_	_	_	FM	9868	FM	33.4 (32.4–34.3)	10.1 (9.40–10.9)	_	_
						M	9421	M	32.3 (31.3–33.2)	10.5 (9.60–11.4)	_	_
						F	447	F	56.8 (52.0–61.4)	9.30 (7.90–10.8)	_	_
Jawadi [43]	2017	Saudi Arabia	_	≥ 18	_	FM	695	FM	48.6 (44.8–52.4)	_	_	_
						M	345		_	_	_	_
						F	350		_	_	_	_
Mahfoud [44]	2015	Qatar	Doha		Observational	FM	2011	FM	72.7 (70.8–74.7)	_	_	_
						M	1885	M	72.5 (70.5–74.5)	_	_	_
						F	126	F	75.4 (66.9–82.6)	_	_	_
Routley [45]	2008	China	Nanjing and Zhoushan	_	Observational	FM	15,2128	FM	49.0 (47.2–50.0)	6.40 (6.00–6.70)	0.40 (0.30–0.50)	_
						M	76,591	M	48.6 (48.1–49.0)	5.60 (5.20–5.90)	0.30 (0.20–0.40)	_
						F	18,697	F	53.2 (51.8–54.5)	8.00 (7.40–8.60)	0.40 (0.20–0.50)	_
Routley [46]	2007	China	Nanjing	_	Observational	FM	31,700	FM	67.3 (66.6–68.0)	19.0 (18.00–19.8)	0.50 (0.30–0.70)	_
						M	24,672	M	67.0 (66.3–67.8)	17.7 (16.6–18.7)	0.60 (0.40–0.90)	_
						F	6678	F	68.9 (66.5–71.2)	21.2 (19.7–22.8)	0.50 (0.20–0.90)	_
Xiao [47]	2017	China	_	≥ 18	Randomly sampled	FM	98,254	FM	7.00 (6.00–8.00)	_	_	Usually
								FM	8.00 (6.00–10.0)	_	_	Sometimes
						M	_	M	8.00 (6.00–10.0)	_	_	Usually
								M	9.00 (7.00–11.0)	_	_	Sometimes
						F	_	F	1.00 (1.00–3.00)	_	_	Usually
								F	0.40 (0.01–1.80)	_	_	Sometimes
Nabipour [48]	2014	Iran	Tehran	_	Observational	FM	10,752	FM	70.9 (70.0–71.7)	_	_	_
						M	9941	M	70.8 (69.9–71.7)	_	_	_
						F	811	F	71.3 (68.0–74.3)	_	_	_
Mohammadi [49]	2009	Iran	Kerman	_	Randomly, observational	FM	800	FM	56.9 (52.6–59.6)	_	_	_
Mohamed [50]	2011	Malaysia	_	Mean 30 ± 9.7	_	FM	793	FM	_	_	22.7 (19.8–25.7)	Always
								FM	_	_	17.1 (14.5–19.9)	Often
								FM	_	_	28.5 (25.3–31.7)	Sometimes
						M	459	M	_	_	44.4 (39.4–49.4)	_
						F	324	F	_	_	46.3 (40.1–52.6)	_
Reagan [51]	2013	USA	_	≥ 18	_	FM	134	FM	61.9 (53.1–70.1)	_	_	Consistent
								FM	20.9 (14.3–28.7)	_	_	Occasional
						M	73	M	60.2 (48.1–71.5)	_	_	Consistent
								M	19.1 (10.9–30.0)	_	_ _	Occasional
						F	61	F	63.9 (50.6–75.8)	_	_	Consistent
								F	22.9 (13.1–35.5)	_	_	Occasional
Martínez-Sánchez [52]	2014	Spain	Barcelona	≥ 18	Observation	FM	2442	FM	89.5 (87.9–90.9)	95.4 (93.5–96.8)	67.6 (63.6–71.4)	_
						M		M	97.6 (96.6–98.4)	_	_	_
						F		F	98.6 (97.2–99.4)	_	_	_
Abu-Zidan [53]	2012	UAE	Al-Ain	_	_	FM	783	FM	25.6 (21.6–30.0)	6.50 (3.40–11.2)	1.30 (0.01–4.80)	_
Afukaar [54]	2010	Ghana	Kumasi Metropolis	_	Observational	FM	11,827	FM	17.6 (16.9–18.2)	4.90 (4.40–5.30)	_	_
						M	11,334	M	16.4 (15.7–17.0)	4.70 (4.20–5.20)	_	_
						F	493	F	44.8 (40.1–49.1)	5.4.0 (4.50–6.30)	_	_
Beck [55]	2009	USA	_	≥ 18	_	FM	347,280	FM	82.4 (82.1–82.7)	_	_	Always
Briggs [56]	2006	USA	Non-Hispanic white	≥ 16	_	FM	67,637	FM	40.3 (39.9–40.8)	44.8 (43.9–45.7)	_	_
			Mexican American					FM	43.7 (42.1–45.3)	43.8 (41.3–46.4)	_	_
			Central American/South American					FM	48.2 (44.4–52.2)	46.8 (40.7–53.0)	_	_
			Puerto Rican					FM	43.1 (34.2–52.5)	39.3 (33.9–44.9)	_	_
			Cuban					FM	39.6 (34.3–45.1)	41.0 (32.0–50.5)	_	_
Sadeghnejad [57]	2014	Iran	Tehran		Stratified multistage, randomly	FM	11,483	FM	77.9 (76.9–78.8)	43.7 (42.1–45.2)	_	_
						M	9334	M	77.5 (76.5–78.4)	44.7 (42.6–46.7)	_	_
						F	2150	F	81.0 (77.9–83.6)	42.1 (39.5–44.7)	_	_
Han [58]	2015	Nebraska	_	≥ 15		FM	10,479	FM	83.0 (82.3–83.8)	_	_	_
						M	4439	M	77.1 (75.8–78.3)	_	_	_
						F	6040	F	87.5 (86.6–88.3)	_	_	_
Kim [59]	2009	USA	_	Teenage	Observational	FM	14,026	FM	60.7 (59.9–61.4)	64.1 (61.6–66.6)	_	_
						M	7620		_	_	_	_
						F	7837		_	_	_	_
Kim [60]	2019	Korea	_	Mean 34.7	_	FM	419	FM	82.8 (76.7–87.9)	64.4 (54.6–73.5)	24.8 (17.5–33.3)	_
						M	247			_	_	_
						F	172			_	_	_
Kwak [61]	2015	Korea	_	≥ 19	_	FM	23,698	FM	76.6 (75.9–77.3)	45.6 (44.6–46.6)	_	_
						M	12,527			_	_	_
						F	11,171			_	_	_
Lardelli-Claret [62]	2009	Spain	_	≥ 18	_	FM	84,338	FM	90.4 (90.2–90.6)	88.8 (88.6–89.1)	_	_
Molnar [63]	2012	USA	_	_	_	FM	19,090	FM	66.1 (65.5–66.8)	_	_	_
						M	13,439	M	62.3 (61.4–63.1)	_	_	_
						F	5651	F	75.3 (74.2–76.5)	_	_	_
Sabzevari [64]	2016	Iran	Kashmar, Bardaskan, Khalilabad		Observational	FM	10,255	FM	51.8 (50.8–52.7)	_	_	_
						M	9798	M	51.4 (50.4–52.3)	_	_	_
						F	457	F	60.5 (55.9–65.1)	_	_	_
Zambon [65]	2008	Italy	Veneto region	_	Multistage sample stratification	FM	16,040		_	_	_	_
					Observational 2003	M		M	_	_	10.6 (9.1–12.1)	_
						F		F	_	_	11.7 (10.4–13.0)	_
									_	_		_
					Self-reported 2003	M		M	_	_	13.5 (12.0–15.1)	_
						F		F	_	_	17.5 (15.8–19.1)	_
									_	_		_
					Observational 2005	M		M	_	_	25.0 (23.0–27.0)	_
						F		F	_	_	27.6 (25.7–29.4)	_
									_	_		_
					Self-reported 2005	M		M	_	_	35.8 (33.2–38.4)	_
						F		F	_	_	38.8 (36.9–40.7)	_
Dulf [66]	2020	Romania	Cluj-Napoca	_	Observational	FM	768	FM	66.8 (63.3–70.1)	_	_	_
						M	469			_	_	_
						F	299			_	_	_
Beck [67]	2019	USA	_	≥ 18	Probability-based sampling	FM	4170	FM	_	86.1 (85.0–87.1)	61.6 (60.0–63.1)	Always
						M	2009	M	_	82.2 (80.5–83.9)	60.0 (57.7–62.3)	
						F	2161	F	_	89.6 (88.4–90.9)	62.9 (60.9–650)	
Bhat [9]	2015	USA	_	≥ 18	_	FM	3953	FM	_		62.0 (60.4–63.5)	Always
						M	1804	M	_		60.0 (56.9–63.1)	
						F	2149	F	_		62.9 (60.1–65.8)	
Boakye [68]	2019	USA	East Tennessee	_	A multistage area probability, observational	FM	33,310	FM	92.0 (91.6–92.3)	85.4 (85.0–85.8)	_	_
						M	22,172	M	86.0 (85.5–86.5)	79.0 (78.2–79.7)	_	_
						F	11,138	F	86.0 (85.3–86.6)	89.0 (88.5–89.4)	_	_
Crandon [69]	2006	Jamaica	Kingston	_	Observational	FM	2846	FM	81.2 (79.5–82.7)	74.0 (70.7–77.1)	_	_
						M	2028	M	77.3 (75.2–79.2)	66.3 (60.8–71.3)	_	_
						F	1014	F	92.5 (90.0–94.4)	80.0 (75.7–83.6)	_	_
Fong [70]	2016	Australia	_	≥ 75	Observational	FM	367	FM	97.0 (94.7–98.4)		_	_
Iribhogbe [71]	2008	Nigeria	Benin		Observational	FM	1785	FM	52.3 (47.0–57.5)	18.4 (14.1–23.3)	6.10 (4.80–7.70)	_
Kamal [72]	2015	Malaysia	Selangor	18–39	Convenience sampling	FM	408	FM	45.1 (40.2–50.0)	_	_	Always
								FM	27.9 (23.6–32.5)	_	_	Most of the time
								FM	20.5 (16.7–24.8)	_	_	Sometimes
										_	_	
						M	184	M	40.2 (33.0–47.6)	_	_	Always
								M	28.2 (21.8–35.3)	_	_	Most of the time
								M	26.0 (19.9–33.0)	_	_	Sometimes
										_	_	
						F	224	F	49.1 (42.3–55.8)	_	_	Always
								F	27.6 (21.9–34.0)	_	_	Most of the time
								F	16.0 (11.5–21.5)	_	_	Sometimes
Febres [73]	2020	Spain	_	_	_	FM	257,851	FM	74.4 (74.2–74.5)	_	_	_
						M	178,839			_	_	
						F	76,837			_	_	
Ünal [74]	2020	Turkey	Semi-rural	Mean 16.0 ± 1.2	_	FM	1465	FM	77.1 (74.9–79.2)	_	_	_
						M	759	M	74.3 (71.0–77.3)	_	_	_
						F	706	F	80.1 (77.0–83.0)	_	_	_
Shaaban1 [75]	2018	Qatar	_	18–25		FM	3049	FM	61.4 (59.4–63.3)	48.8 (44.5–53.2)	_	_
						M	1856	M	58.0 (55.6–60.4)	40.6 (33.6–47.8)	_	_
						F	1193	F	67.9 (64.7–71.0)	53.6 (48.2–59.0)	_	_
Shaaban [76]	2020	Qatar	Doha	≥ 18	_	FM	7908	FM	83.6 (82.7–84.4)	_	_	_
						M	7180	M	83.3 (82.4–84.2)	_	_	_
						F	728	F	86.1 (83.4–88.5)	_	_	_
Siddiqui [77]	2014	Pakistan	Karachi	_	convenience	FM	212	FM	15.0 (10.5–20.6)	_	_	Regularly
						M	126	FM	34.4 (28.0–41.2)	_	_	Occasionally
						F	86			_	_	
Li [78]	2018	China	Shanghai	_	Stratified, observational	FM	77,641	FM	88.4 (88.1–88.6)	_	_	_
						M	61,561		_	_	_	_
						F	61,088		_	_	_	_
Kulanthayan [79]	2004	Malaysia	_	_	_	FM	273	FM	76.6 (68.6–83.4)	56.0 (41.2–70.0)	_	_
Lerner [80]	2001	USA	_	13–93	_	FM	1656	FM	71.9 (69.7–74.1)	_	_	_

### Statistical analysis

The pooled prevalence of wearing a seat belt with a 95% confidence interval was calculated using random-effect meta-analyses. Inter-study heterogeneity was assessed using chi-squared tests and the *I*^2^ statistic. Additionally, subgroup analyses were performed to explore the sources of heterogeneity, and Egger’s test was used to detect publication bias. The STATA-v14 (Stata Corp, TX, USA) software was used to analyze the data [81].

## Results

**Fig. 1:**
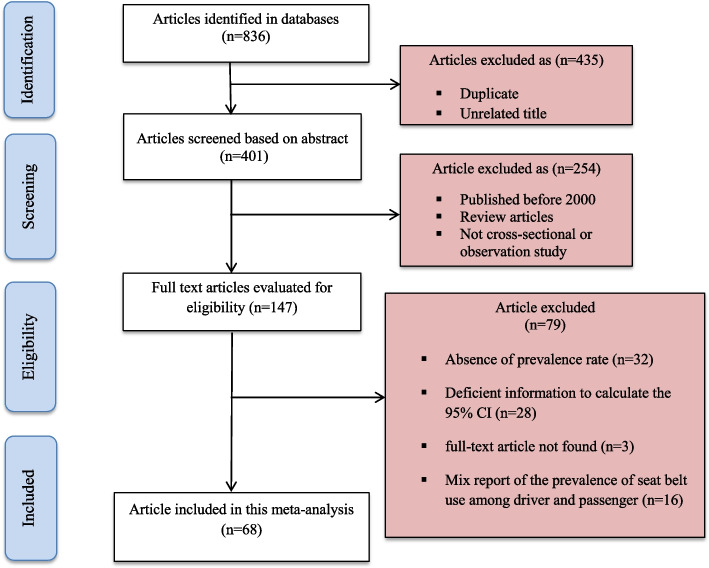
PRISMA flowchart of the study selection process

Out of 836 identified articles in the databases, 435 were excluded due to duplication or unrelated titles. Another 254 articles were removed after screening based on the abstract as they were review articles, published before 2000, or were not cross-sectional or observation studies. After that, 147 full-text articles were reviewed and assessed according to the eligibility criteria. Out of these, 79 articles were excluded as they did not report the prevalence or had sufficient data to calculate 95% CI or had mixed reporting of the prevalence of seat belt use among drivers and passengers. Finally, 68 articles with a total of 1,490,226 participants that met the inclusion criteria were included in this meta-analysis. The flowchart of the study selection process is shown in Fig. 1.

Out of 68 studies included in the meta-analysis, ten were considered high quality, 53 were considered moderate quality, and five were considered low quality (Supplementary Table S1). The pooled prevalence of seat belt use among drivers in the high-quality studies, the moderate quality studies, and the low-quality studies were 37.12% (95% *CI*: 33.00–41.76), 47.72% (95% *CI*: 45.77–49.75), and 37.75% (95% *CI*: 27.46–51.91), respectively, which showed some evidence of heterogeneity (*P* < 0.001) in terms of study quality. Therefore, we estimated pooled prevalence based on the type of vehicle in the three study groups, and no heterogeneity was observed. Therefore, the quality of the studies did not affect the present meta-analysis results (Supplementary Table S2).

### Prevalence of seat belt use

In general, the pooled prevalence of seat belt use among drivers, front-seat passengers, and rear-seat passengers was 43.94% (95% *CI*: 42.23–45.73), 38.47% (95% *CI*: 34.89–42.42), and 15.32% (95% *CI*: 12.33–19.03), respectively. The lowest prevalence of seat belt use among drivers was in Asia [37.86% (95% *CI*: 34.44–41.61)] and Middle East [38.17% (95% *CI*: 34.25–42.55)] region, and the highest was in Western Europe [84.42% (95% *CI*: 72.32–98.55)] and the Americas [51.57% (95% *CI*: 47.54–55.93)]. Also, the lowest prevalence of seat belt use among front- and rear-seat passengers was found in Africa (17.43%, 2.29%), Asia (34.62%, 7.93%), and the Middle East (31.53%, 9.24%) (*P* < 0.001).

The prevalence of seat belt use was significantly higher in female drivers [51.47% (95% *CI*: 48.62–54.48)] compared to male drivers [38.27% (95% *CI*: 34.98–41.87)] (*P* < 0.001). In addition, the prevalence of seat belt use among female front-seat passengers [33.09% (95% *CI*: 29.45–37.18)] and rear-seat passengers [18.27% (95% *CI*: 13.73–24.32)] was higher than among male front-seat passengers [25.96% (95% *CI*: 20.18–33.38)] and rear-seat passengers [15.55% (95% *CI*: 10.97–22.04)], although this difference was not statistically significant (*P* = 0.08, *P* = 0.48).

The drivers and front-seat passengers of SUVs (68.9%, 50.5%) and vans (70.39%, 19.83%) showed a higher prevalence of seat belt use compared to drivers and front-seat passengers of other vehicle types. In contrast, drivers of buses (21.84%) and minibuses (28.16%) and front-seat passengers of minibuses (1.80%) and taxis (3.82%) displayed lower prevalence of seat belt use (*P* < 0.001).

Furthermore, the highest prevalence of seat belt use among drivers was in the morning [54.89% (95% *CI*: 46.85–64.32)], followed by in the afternoon/evenings [50.78% (95% *CI*: 43.52–59.25)] and the night [46.59% (95% *CI*: 31.49–68.91)], but the differences were not statistically significant (*P* = 0.66). On the other hand, the highest prevalence of seat belt use among front-seat passengers was at night [51.3% (95% *CI*: 41.80–60.70)] (*P* < 0.001).

**Fig. 2:**
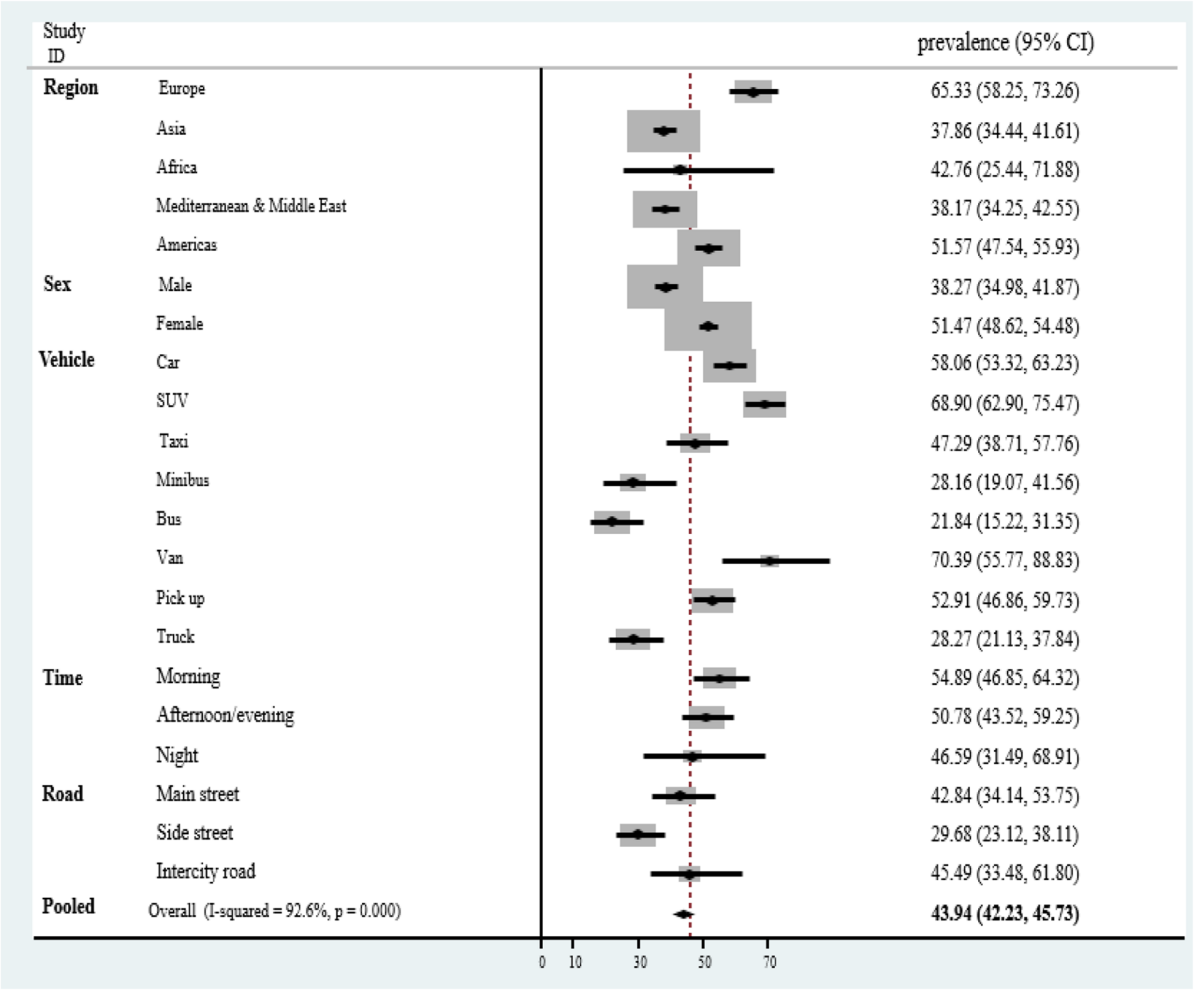
Sensitivity analysis of the prevalence of seat belt usage among drivers

In addition, the highest prevalence of seat belt use among drivers was observed on intercity roads [45.49% (95% *CI*: 33.48–61.80)], while the lowest prevalence was observed on side streets [29.68% (95% *CI*: 23.12–38.11)] (*P* = 0.04). Additionally, the highest prevalence of seat belt use among front-seat passengers was also on intercity roads [16.98% (95% *CI*: 4.06–70.91)] (*P* = 0.28) (Table 2, Fig. 2).

**Table 2:** The prevalence of seat belt use among drivers and passengers

Variables	No. of studies (population)	Driver			Front seat passengers			Rear seat passenger		
		NR	Prevalence (95% *CI*)	Test for heterogeneity (*p*-value)	NR	Prevalence (95% *CI*)	Test for heterogeneity (*p*-value)	NR	Prevalence (95% *CI*)	Test for heterogeneity (*p*-value)
**Total**	68 (1,490,226)	86	43.94 (42.23–45.73)		36	38.47 (34.89–42.42)		21	15.30 (12.33–19.03)	
**Region**										
Europe	5 (361,207)	6	65.33 (58.25–73.26)	< 0.001	4	65.61 (57.93–74.32)	< 0.001	11	22.86 (16.40–31.85)	< 0.001
Asia	16 (417,770)	24	37.86 (34.44–41.61)		6	34.62 (16.97–70.65)		8	7.930 (3.340–18.82)	
Africa	7 (32,750)	7	42.76 (25.44–71.88)		6	17.43 (6.580 – 46.12)		3	2.290 (0.820 – 6.370)	
Mediterranean & Middle East	19 (58,081)	26	38.17 (34.25–42.55)		8	31.53 (23.58–42.16)		2	9.24 (0.460–183.23)	
Americas	21 (619,012)	27	51.57 (47.54–55.93)		13	64.92 (60.29–69.90)		4	64.44 (60.90–68.19)	
**Sex**										
Male	39 (499,866)	43	38.27 (34.98–41.87)	< 0.001	12	25.96 (20.18–33.38)	0.08	13	15.55 (10.97–22.04)	0.48
Female	39 (232,169)	43	51.47 (48.62–54.48)		12	33.09 (29.45–37.18)		13	18.27 (13.73–24.32)	
**Type of vehicle**										
Car	19 (405,955)	20	58.06 (53.32–63.23)	< 0.001	8	24.25 (15.17–38.76)	< 0.001	3	3.270 (00.15–71.33)	
SUV	6 (130,171)	7	68.90 (62.90–75.47)		1	50.50 (44.60–56.30)		**_**	**_**	
Taxi	10 (314,269)	10	47.29 (38.71–57.76)		5	3.820 (0.830–17.52)		2	0.200 (00.13–0.300)	
Minibus	6 (129,070)	6	28.16 (19.07–41.56)		2	1.800 (00.38–08.49)		**_**	**_**	
Bus	7 (112,558)	7	21.84 (15.22–31.35)		4	4.030 (2.690–6.040)		**_**	**_**	
Van	6 (240,893)	6	70.39 (55.77–88.83)		3	19.83 (7.720–50.89)		1	0.200 (0.001–01.00)	
Pickup	11 (367,358)	11	52.91 (46.86–59.73)		3	9.01 (02.04–39.69)		**_**	**_**	
Truck	10 (134,048)	11	28.27 (21.13–37.84)		4	8.660 (03.26–22.97)		**_**	**_**	
**Time**										
Morning	14 (247,090)	21	54.89 (46.85–64.32)	0.66	9	11.47 (6.820–19.30)	< 0.001	6	0.400 (0.250–0.660)	1.00
Afternoon/evening	12 (243,907)	20	50.78 (43.52–59.25)		8	10.78 (7.640–15.20)		6	0.400 (0.300–0.530)	
Night	5 (18,832)	5	46.59 (31.49–68.91)		1	51.30 (41.80–60.70)		**_**	**_**	
**Type of road**										
Main street	10 (247,122)	10	42.84 (34.14–53.75)	0.04	5	10.78 (6.100–19.04)	0.28	2	0.920 (0.540–1.560)	0.01
Side street	9 (233,400)	9	29.68 (23.12–38.11)		5	6.260 (3.430–11.43)		2	0.270 (0.120–0.590)	
Intercity road	7 (68,729)	8	45.49 (33.48–61.80)		3	16.98 (4.060–70.91)		**_**	**_**

Moreover, a subgroup analysis was performed between the data obtained from national surveys and the findings from observational studies. There was no evidence of heterogeneity between the pooled prevalence of seat belt use among drivers in observational studies [43.26% (95% *CI*: 40.93–45.72)] and national surveys [42.35% (95% *CI*: 38.7–46.24)] (*P* = 0.69).

## Discussion

This study assessed the prevalence of seat belt use among drivers, front-seat passengers, and rear-seat passengers between 2000 and 2020. The results showed that the prevalence of seat belt use among drivers was not high (43.94%). Additionally, the study found that rear-seat passengers (15.32%) are less likely to always or more often wear seat belts compared to front-seat passengers (38.47%), which is consistent with the results of other studies [67, 82, 83]. A survey of adults also revealed that those who did not wear seat belts in the back seat believed that the rear seat was safer than the front seat and that seat belts were not necessary on short trips [35].

This study showed that the prevalence of seat belt use in the Asian, Mediterranean and Middle East, and African regions was lower than in Europe and the Americas, which may partially be attributed to the differences in driving laws and regulations within countries in these regions.

According to Word Health Organization’s previous reporting, the African region has the highest traffic injury death rates, while the European region has the lowest [2]. Other studies have also highlighted that the prevalence of seat belt use is not high in low- and middle-income countries, and more than 90% of the world’s road fatalities occur in these countries [7, 84].

The National Highway Traffic Safety Administration (NHTSA) of the USA recommended that wearing a seat belt is one of the safest choices that drivers and passengers can make on the road. It also asserted that the national use rate was 90.4% in 2021, and that seat belts saved an estimated 14,955 American lives in 2017 alone, and they could have saved an additional 2549 people if they had been wearing seat belts [85].

It has been demonstrated that in 2013, almost 85,000 people died from road traffic injuries in the WHO European Region. In this region, 95% of the population is governed by comprehensive traffic laws which are in line with best practices for seat belts. Additionally, in 36 European countries, the median reported usage of seat belts among front-seat occupants was 86%, and the median proportion of rear seat-belt use was 65% [86].

This study also found that the prevalence of seat belt use in women drivers and passengers was significantly higher than in men. This pattern has been observed in other studies [87–89], which may be due to women being more conservative drivers and more likely to follow driving laws. Previous studies have also reported more traffic violations among men than women [90], which explains why men are more likely to be injured in traffic accidents [23].

This study observed a significant relationship between the type of vehicle and seat belt use among drivers and passengers. SUV drivers and passengers were more likely to wear seat belts than drivers and passengers of other vehicles, which is consistent with a study conducted in the USA [91]. This difference could be due to various factors, including SUV drivers and passengers exhibiting healthier behaviors due to their higher socioeconomic status [92, 93].

On the other hand, drivers and passengers of public vehicles (buses, minibuses, and taxis) tend to wear seat belts less frequently. In many countries, public transport makes frequent stops, and drivers are sometimes forced to disembark at many stations to meet passengers’ needs, making it uncomfortable for them to wear seat belts. Other studies have also shown that seat belt use is less common in public transport due to the frequent stops [71, 94].

Previous studies have shown a statistically significant relationship between seat belt use and the time of day [37, 95, 96]. In many countries, officers are usually present to monitor the roads at any time of day, and drivers familiar with the regulations tend to use seat belts as a precaution. In agreement, this study found that drivers wore seat belts more frequently during the day than at night, although this difference was not statistically significant. This study also showed that drivers were more likely to use seat belts while driving on intercity roads than on the main and side streets in the city. This finding may be attributed to the greater presence of traffic police and the higher number of traffic cameras on intercity roads. Moreover, a study in Nigeria has shown that seat belt use is more common on interurban roads than on rural roads [97].

### limitations

Finally, there were limitations to this study that should be taken into consideration when interpreting the results. The first limitation was the different methods used to measure seat belt usage across different studies. The second limitation was the unequal number of studies conducted in the five geographical regions, as well as the use of different sampling methods, which could be a contributing source to the variation in the prevalence of seat belt use across these regions. The third limitation was the lack of information on seat belt use by time and road type in some studies.

### Conclusion

This meta-analysis showed that, in general, the prevalence of seat belt use among drivers and car passengers is not high. Seat belt use was found to be lower in Asia, the Mediterranean and Middle East, and Africa compared to Europe and the Americas. We also found that women wore seat belts significantly more than men. Furthermore, seat belt use among drivers and passengers of public transportation (buses, minibuses, taxis) was lower than in other vehicles. Therefore, it is necessary to design and implement well-structured targeted interventional programs, such as developing training campaigns about the benefits of seat belt use among these vulnerable populations, especially in Asia, the Mediterranean and Middle East, and Africa. Additionally, we recommend further research be conducted to explore the factors that affect drivers’ and passengers’ attitudes and knowledge about seat belt use.

### Supplementary Information

**Additional file 1: Table S1.** Quality assessment for included studies. **Table S2.** The prevalence of seat belt use based on the type of vehicle in three studies groups.

## Data Availability

Data are available upon request.

## Abbreviations

SUVs: Sports utility vehicles
GDP: Global gross domestic product
