# Supplementary material for: The prevalence of seat belt use among drivers and passengers: a systematic review and meta-analysis
Source: J Egypt Public Health Assoc. 2023 Aug 2;98:14. doi: 10.1186/s42506-023-00139-3 (PMC10393920; doi:10.1186/s42506-023-00139-3)
Supplement: Supplementary file 1 — Additional file 1: Table S1. Quality assessment for included studies. Table S2. The prevalence of seat belt use based on the type of vehicle in three studies groups. [file 42506_2023_139_MOESM1_ESM.docx]

**Table S1: quality assessment for included studies in systematic review and meta-analysis.**

|  | Authors | Year | Country | **Study Design and Sampling Method** | **Sampling Frame** | **Sample Size** | **Appropriate Measurement** | **Unbiased Measurement** | **Response Rate** | **interpretation of the results** | **application of findings** | **Quality score** |
| --- | --- | --- | --- | --- | --- | --- | --- | --- | --- | --- | --- | --- |
| 1 | Sungyop Kim | 2003 | hawaii | 0 | 1 | 1 | 1 | 1 | 1 | 0 | 1 | 6 |
| 2 | Quang Ngoc La | 2013 | Hanoi, Vietnam | 0 | 1 | 1 | 1 | 0 | 1 | 0 | 1 | 5 |
| 3 | Virginia Routley | 2009 | China | 1 | 1 | 1 | 1 | 1 | 1 | 1 | 1 | 8 |
| 4 | GhorbanaliMohammadi | 2011 | Iran | 0 | 1 | 0 | 1 | 0 | 1 | 0 | 1 | 4 |
| 5 | Hannah K. Allena | 2019 | Virginia | 1 | 1 | 1 | 1 | 0 | 1 | 1 | 1 | 7 |
| 6 | Ricardo Pérez-Núñez | 2013 | Mexico | 1 | 1 | 1 | 1 | 1 | 1 | 1 | 1 | 8 |
| 7 | J. Felipe García-España | 2012 | United States | 1 | 1 | 1 | 0 | 0 | 1 | 1 | 1 | 6 |
| 8 | Guang X. Chen, | 2015 | United States | 1 | 1 | 1 | 0 | 0 | 1 | 1 | 1 | 6 |
| 9 | A. Bener | 2013 | Qatar | 1 | 1 | 1 | 0 | 0 | 1 | 0 | 1 | 5 |
| 10 | George Kritsotakis | 2019 | Greece | 1 | 1 | 1 | 0 | 0 | 1 | 0 | 1 | 5 |
| 11 | Mohammadi | 2015 | Iran | 1 | 1 | 1 | 0 | 0 | 1 | 0 | 1 | 5 |
| 12 | Popoola SO | 2013 | Nigeria | 0 | 1 | 1 | 0 | 0 | 1 | 0 | 1 | 4 |
| 13 | O Sangowawa | 2010 | Nigeria | 1 | 1 | 1 | 1 | 0 | 1 | 0 | 1 | 6 |
| 14 | Mohammadzadeh | 2014 | Iran | 0 | 1 | 1 | 0 | 0 | 1 | 0 | 1 | 4 |
| 15 | Ballem Praveen | 2020 | India | 1 | 1 | 1 | 0 | 0 | 1 | 0 | 1 | 5 |
| 16 | A. Bener | 2008 | Qatar | 1 | 1 | 1 | 0 | 0 | 1 | 0 | 1 | 5 |
| 17 | Nathaniel C. Briggs | 2008 | U.S | 1 | 1 | 1 | 1 | 0 | 1 | 1 | 1 | 7 |
| 18 | William G Fernandez | 2006 | USA | 1 | 1 | 1 | 0 | 0 | 1 | 0 | 1 | 5 |
| 19 | A.H Alomari | 2020 | jordan | 0 | 1 | 1 | 1 | 0 | 1 | 0 | 1 | 5 |
| 20 | Rediet Fikru Gebresenbet | 2019 | Ethiopia | 1 | 1 | 0 | 1 | 1 | 1 | 1 | 1 | 7 |
| 21 | Sudha R. Raman | 2013 | Kuwait | 0 | 1 | 1 | 1 | 0 | 1 | 0 | 1 | 5 |
| 22 | Jessica S. Jermakian | 2017 | U.S | 0 | 1 | 1 | 1 | 0 | 1 | 1 | 1 | 6 |
| 23 | Parviz A. Koushki | 2006 | Kuwait | 1 | 1 | 1 | 0 | 0 | 1 | 0 | 1 | 5 |
| 24 | Ting Hway Wong | 2016 | asia | 1 | 1 | 1 | 1 | 0 | 1 | 0 | 1 | 6 |
| 25 | Michael G. Vaughn | 2012 | United States | 1 | 1 | 1 | 1 | 0 | 1 | 1 | 1 | 7 |
| 26 | Nandi L. Taylor | 2019 | USA | 1 | 1 | 1 | 0 | 0 | 1 | 0 | 1 | 5 |
| 27 | Sedigheh Sadat Tavafian | 2011 | iran | 1 | 0 | 0 | 0 | 0 | 1 | 0 | 1 | 3 |
| 28 | Penprapa Siviroj | 2012 | Thailand. | 1 | 1 | 1 | 0 | 0 | 1 | 0 | 1 | 5 |
| 29 | Solomon Ntow Densu | 2013 | GHANA | 0 | 1 | 1 | 0 | 0 | 1 | 1 | 1 | 5 |
| 30 | Ayman H. Jawadi | 2017 | Saudi Arabia | 0 | 1 | 1 | 0 | 0 | 1 | 0 | 1 | 4 |
| 31 | Ziyad R. Mahfoud | 2015 | Qatar | 0 | 1 | 1 | 0 | 0 | 1 | 1 | 1 | 5 |
| 32 | Virginia Routley | 2008 | China | 1 | 1 | 1 | 1 | 0 | 1 | 0 | 1 | 6 |
| 33 | V Routley | 2007 | China | 1 | 1 | 1 | 1 | 0 | 1 | 0 | 1 | 6 |
| 34 | Deng Xiao | 2017 | China | 1 | 1 | 1 | 1 | 0 | 1 | 1 | 1 | 7 |
| 35 | Amir Reza Nabipour | 2014 | Iran | 1 | 1 | 1 | 1 | 0 | 1 | 0 | 1 | 6 |
| 36 | G. Mohammadi | 2009 | Iran | 1 | 1 | 1 | 1 | 0 | 1 | 0 | 1 | 6 |
| 37 | Norlen Mohamed | 2011 | Malaysia | 1 | 1 | 1 | 0 | 0 | 1 | 0 | 1 | 5 |
| 38 | Ian J. Reagan | 2013 | United States | 1 | 1 | 0 | 0 | 0 | 1 | 0 | 1 | 4 |
| 39 | Jose M Martínez-Sánchez | 2014 | Spain | 1 | 1 | 1 | 0 | 0 | 1 | 0 | 1 | 5 |
| 40 | Fikri M. | 2012 | UAE | 1 | 1 | 1 | 0 | 0 | 1 | 0 | 1 | 5 |
| 41 | FRANCIS K | 2010 | Ghana | 0 | 1 | 1 | 0 | 0 | 1 | 0 | 1 | 4 |
| 42 | Laurie F. Beck | 2009 | USA | 1 | 1 | 1 | 1 | 0 | 1 | 1 | 1 | 7 |
| 43 | N C Briggs | 2006 | USA | 1 | 1 | 1 | 1 | 0 | 1 | 1 | 1 | 7 |
| 44 | F. Sadeghnejad | 2014 | Iran | 1 | 1 | 1 | 0 | 0 | 1 | 0 | 1 | 5 |
| 45 | Guang-Ming Han | 2015 | Nebraska | 0 | 1 | 1 | 0 | 0 | 1 | 0 | 1 | 4 |
| 46 | Sungyop Kim | 2009 | USA | 1 | 1 | 1 | 0 | 0 | 1 | 0 | 1 | 5 |
| 47 | Sue Min Kim | 2019 | Korea | 1 | 1 | 1 | 0 | 0 | 1 | 0 | 1 | 5 |
| 48 | Bong Hun Kwak | 2015 | Korea | 1 | 1 | 1 | 0 | 0 | 1 | 0 | 1 | 5 |
| 49 | Pablo Lardelli-Claret | 2009 | Spain | 1 | 1 | 1 | 0 | 0 | 1 | 0 | 1 | 5 |
| 50 | Lisa J. Molnar | 2012 | United States | 1 | 1 | 1 | 0 | 0 | 1 | 0 | 1 | 5 |
| 51 | Javad Torkamannejad Sabzevari | 2016 | Iran | 1 | 1 | 1 | 0 | 0 | 1 | 0 | 1 | 5 |
| 52 | Francesco Zambon | 2008 | taly | 1 | 1 | 1 | 0 | 0 | 1 | 1 | 1 | 6 |
| 53 | Diana Dulf | 2020 | Romania | 1 | 1 | 1 | 0 | 0 | 1 | 0 | 1 | 5 |
| 54 | Laurie F. Beck | 2019 | USA | 1 | 1 | 1 | 1 | 0 | 1 | 1 | 1 | 7 |
| 55 | Geeta Bhat | 2015 | USA | 1 | 1 | 1 | 1 | 0 | 0 | 1 | 1 | 6 |
| 56 | Kwaku F. Boakye | 2019 | USA | 1 | 1 | 1 | 1 | 0 | 1 | 0 | 1 | 6 |
| 57 | IW Crandon | 2006 | Jamaica | 1 | 1 | 1 | 0 | 0 | 1 | 0 | 1 | 5 |
| 58 | Cameron k. Fong | 2016 | Australia | 0 | 1 | 1 | 0 | 0 | 1 | 0 | 1 | 4 |
| 59 | Pius Ehiawaguan Iribhogbe | 2008 | Nigeria | 1 | 1 | 1 | 0 | 0 | 1 | 0 | 1 | 5 |
| 60 | Ahmad Kamal | 2015 | Malaysia | 0 | 0 | 1 | 0 | 0 | 1 | 0 | 1 | 3 |
| 61 | Juan Diego Febres | 2020 | Spain | 0 | 1 | 1 | 1 | 0 | 1 | 0 | 1 | 5 |
| 62 | [E Ünal](https://scholar.google.com/citations?user=mAV9XSIAAAAJ&hl=en&oi=sra) | 2020 | Turkey | 0 | 1 | 1 | 1 | 0 | 0 | 0 | 0 | 3 |
| 63 | Khaled Shaaban1 | 2018 | Qatar | 1 | 1 | 1 | 1 | 0 | 1 | 0 | 1 | 6 |
| 64 | Khaled Shaaban | 2020 | Qatar | 0 | 1 | 1 | 1 | 0 | 1 | 0 | 1 | 5 |
| 65 | Emaduddin Siddiqui | 2014 | Pakistan | 0 | 0 | 0 | 1 | 0 | 0 | 0 | 1 | 2 |
| 66 | Qingfeng Li | 2018 | China | 1 | 1 | 1 | 1 | 0 | 1 | 0 | 1 | 6 |
| 67 | [S Kulanthayan](https://scholar.google.com/citations?user=ct_QSh4AAAAJ&hl=en&oi=sra) | 2004 | Malaysia | 0 | 1 | 0 | 1 | 0 | 0 | 0 | 1 | 3 |
| 68 | E. Brooke Lerner | 2001 | USA | 0 | 1 | 1 | 1 | 0 | 1 | 0 | 1 | 5 |

Table S2: The prevalence of seat belt use based on the type of vehicle in three studies groups.

| variables | High-quality study | Medium quality study | Low-quality study | **p-value** |
| --- | --- | --- | --- | --- |
| car | 46.51 (30.0 – 72.10) | 52.44 (43.28 – 63.54) | 48.03 (31.46 – 73.32) | =0.84 |
| SUV | 50.63 (27.61 – 92.82) | 57.20 (39.91 – 81.99) | 51.90 (28.68 – 93.91) | =0.92 |
| taxi | 41.31 (32.63 – 52.29) | 47.70 (45.79 – 49.68) | 43.63 (35.19 – 54.10) | =0.37 |
| minibus | 34.50 (27.16 – 43.81) | 38.04 (22.80 – 63.46) | 33.27 (25.17 – 43.98) | =0.90 |
| bus | 29.30 (17.47 – 49.12) | 32.98 (15.35 – 70.86) | 28.84 (16.95 – 49.07) | =0.95 |
| van | 50.69 (27.08 – 94.89) | 56.94 (38.97 – 83.22) | 51.93 (28.07 – 96.05) | =0.93 |
| pickup | 44.30 (31.30 – 62.70) | 49.43 (44.90 – 54.41) | 46.07 (33.15 – 64.04) | =0.78 |
| truck | 33.51 (25.87 – 43.39) | 37.49 (22.48 – 62.53) | 32.34 (24.48 – 42.72) | =0.88 |
